# High-resolution short-exposure small-animal laboratory x-ray phase-contrast tomography

**DOI:** 10.1038/srep39074

**Published:** 2016-12-13

**Authors:** Daniel H. Larsson, William Vågberg, Andre Yaroshenko, Ali Önder Yildirim, Hans M. Hertz

**Affiliations:** 1 Department of Applied Physics, KTH Royal Institute of Technology/Albanova, 106 91 Stockholm, Sweden; 2 Physik-Department & Institut für Medizintechnik, Technische Universität München, Garching, Germany; 3 Institute of Lung Biology and Disease, Member of the German Center for Lung Research (DZL), Helmholtz Zentrum München, Neuherberg, Germany

## Abstract

X-ray computed tomography of small animals and their organs is an essential tool in basic and preclinical biomedical research. In both phase-contrast and absorption tomography high spatial resolution and short exposure times are of key importance. However, the observable spatial resolutions and achievable exposure times are presently limited by system parameters rather than more fundamental constraints like, e.g., dose. Here we demonstrate laboratory tomography with few-ten μm spatial resolution and few-minute exposure time at an acceptable dose for small-animal imaging, both with absorption contrast and phase contrast. The method relies on a magnifying imaging scheme in combination with a high-power small-spot liquid-metal-jet electron-impact source. The tomographic imaging is demonstrated on intact mouse, phantoms and excised lungs, both healthy and with pulmonary emphysema.

X-ray computed tomography (CT) of small animals and organs is extensively used in preclinical medical research for understanding mechanisms of disease, drug development and phenotyping^1^,^2^. However, present small-animal CT systems typically exhibit limited spatial resolution and/or long exposure times. Phase methods provide larger contrast in many imaging situations and thereby a path to lower dose and shorter exposure times while still keeping the spatial resolution high^3^. Here we demonstrate that phase-contrast as well as absorption-contrast 3D imaging can be performed with high spatial resolution and short exposure times at acceptable dose by the combination of a magnifying propagation-based arrangement and a high-power liquid-metal-jet micro-focus x-ray source.

Micro-CT of small animals is a cost-effective method that provides detailed 3D morphological imaging with high throughput^2^. The key imaging parameters are spatial resolution, scan time and dose. The linear spatial resolution of micro-CT should be more than 10× higher than that of clinical CT (i.e., <100 μm) due to the smaller scale of organs and other structures in, e.g., a rodent^4^. Any additional improvement in resolution shows promise to reveal significant information of medical relevance. The total scan times should be kept short in order to avoid excessive periods of anesthesia, which may modify results and hamper longitudinal *in-vivo* studies. Furthermore, short exposure times lessen the influence of sample movement on the spatial resolution. Finally, the dose should be low enough to be acceptable for *in-vivo* studies. For a typical few-minute scan with a few-100-mGy dose, present micro-CT systems generally are limited to few-100 μm observable resolution also when absorption contrast is high (e.g., for bone imaging)^2^. Clearly, many imaging applications rely on lower absorption contrast resulting in much lower observable spatial resolution or unacceptably long scan times and/or high dose.

Phase-contrast x-ray imaging methods have demonstrated improved contrast in, e.g., soft tissue^3^,^5^. The higher contrast can, e.g., be exploited for higher-resolution imaging while still keeping dose and/or scan time acceptable. There are several x-ray phase-contrast imaging methods suitable for synchrotron sources^3^ but only grating-based imaging (GBI)^6^, and propagation-based imaging (PBI)^7^,^8^ have been widely used with laboratory sources for bioimaging. GBI basically detects the gradient of the phase shift (∇ϕ) and provides quantitative data on absorption, phase shift and small-angle scatter via serial measurements (phase stepping of the gratings). PBI typically detects the transverse Laplacian of the phase (∇^2^ϕ) with a single measurement and phase retrieval is necessary for quantitative interpretation. Although the intrinsic quantitative nature of GBI is an advantage for tomographic imaging, the extra optical elements (gratings) in the GBI arrangement typically cause a dose and exposure-time disadvantage compared to the free-space propagation of PBI. There are only few comparisons on observable detail vs dose for the two methods but for imaging gas-filled structures (like CO_2_-filled blood vessels or air-filled lung alveoli) the necessary dose for observing sub-50-μm structures may differ a factor ten in favor of PBI^9^. Given that laboratory systems are typically limited by source power, this factor ten directly translates also into a shorter exposure time. Although this dose and exposure-time advantage of PBI over GBI is not a general result and the performance of the methods must be evaluated for each individual imaging task, the present study was performed with PBI since it has demonstrated lower-dose and shorter-exposure-time high-resolution imaging of the specific class of objects (gas-tissue interfaces) discussed here.

Any small-animal phase-contrast imaging system aiming for high spatial resolution benefits from a high-brilliance source. Consequently much of the early as well as present phase-contrast imaging was and is performed at synchrotron facilities^3^. However, small-animal imaging is typically an integral part of other investigations making it beneficial to have a laboratory imaging system in-house. A dedicated GBI-based tomographic scanner based on a classical microfocus source^10^ has demonstrated impressive rodent imaging^11^. Still, the microfocus source of this system lacks sufficient brightness and spot size to allow high-spatial-resolution imaging with reasonable scan times. Several laser-/accelerator-based “compact” systems have been proposed as alternative high-brightness laboratory sources^12^,^13^,^14^. One of these, the inverse-Compton scattering Compact Light Source (CLS), has achieved sufficient stability for high-quality tomographic imaging, both in absorption, where sub-100 μm bone imaging has been demonstrated with approx. 20 minutes exposure time^14^,^15^, and in grating-based phase-contrast tomography, where 80-μm resolution was reached with several hours of exposure time^16^. Although the accelerator-based compact sources appears to have potential for improvement in brightness, their complexity and size makes it difficult to envision them on a rotating gantry.

In addition to the immediate small-animal-imaging applications discussed above, absorption- and phase-contrast tomography on excised samples is presently emerging as an alternative to conventional destructive histology^17^,^18^. The advantages include speed, 3D with thinner effective slicing, and less destructive and simplified sample preparation. Also in this application high spatial resolution and short exposure times are essential while dose is of lesser concern. With a short exposure time the risk of sample movement decrease, which is vital for obtaining the high resolution important in most histological analyses. Present systems for high-spatial-resolution phase-contrast virtual histology typically use hours of exposure time at synchrotrons^17^,^18^.

Here we demonstrate that laboratory x-ray tomography can be performed with minute exposure times and few-10-μm resolution, both in absorption-contrast bone imaging and in phase-contrast soft tissue imaging. The method relies on a magnifying propagation-based arrangement in combination with a high-brightness liquid-metal-jet electron-impact source^19^. This source type has previously demonstrated its applicability for very high spatial resolution (cellular and sub-cellular) phase-contrast imaging of blood vessels, tumors, lung tissue, and muscle tissue in organs and in whole-body mouse and zebrafish, but with long exposure times and high dose^20^,^21^,^22^,^23^. In the present paper our imaging system is optimized to allow observation of high-spatial resolution features (few-10 μm range) at reasonably short scan times (few-minute range) and at a dose acceptable for *in-vivo* rodent imaging (few-100 mGy range). We demonstrate the system for bone absorption imaging in intact mouse and for phase-contrast imaging of phantoms and excised mouse lungs, both healthy and with pulmonary emphysema.

## Results

### Laboratory x-ray tomography with short exposure time and high resolution

**Figure 1:**
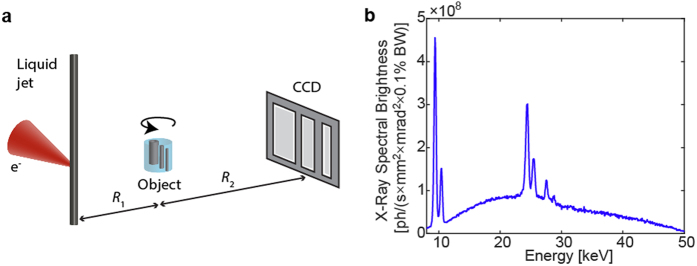
Laboratory propagation-based imaging with short exposure times and high spatial resolution. (**a**) The small-spot high-brightness liquid-metal-jet microfocus source illuminates the object in a magnifying scheme. Depending on the setting of source-object-distance (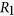) and the object-detector distance (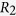) the contrast can be tuned from pure absorption to phase-contrast (PBI). The object is rotated around its vertical axis for the tomography. (**b**) The emitted Al-filtered spectrum consists of line emission from Ga (Kα at ∼9 keV) and In (Kα ∼24 keV) as well as a bremsstrahlung background. We use a 210 μm Al filter to reduce low-energy emission that else would contribute to excessive dose via absorption in the object.

Figure 1a depicts the experimental arrangement. This is a classical magnifying x-ray tomography arrangement with a microfocus x-ray source, a sample, and a detector. The magnification *M* = *(R*_1_ + *R*_2_*)/R*_1_ allows high resolution imaging also beyond the limitation set by detector resolution, provided the source spot-size is small. By changing the effective propagation distance *z*_eff_ = *(R*_1_*R*_2_*)/(R*_1_ + *R*_2_) the contrast can be tuned from pure absorption (short *z*_eff_) to increasing phase contrast (longer *z*_eff_ resulting in propagation-based phase imaging, PBI)^24^, albeit typically at the price of longer exposure times. Thus, the arrangement allows for quick and simple adaption to optimize imaging properties (e.g., resolution and contrast) to the studied sample. Both absorption and phase-contrast imaging is illustrated below. However, for the arrangement to provide proper contrast, resolution and exposure times, it critically relies on the microfocus source properties.

For whole-body imaging of, e.g., a mouse we need a high-brightness microfocus source operating at a few tens of kV. We employ a 50 kV liquid-metal-jet source operating at 400 W electron-beam power with a spot size of 8 μm. The high power is critical for the short exposure times and the small spot size provides high spatial resolution as well as the spatially coherent illumination necessary for high contrast in PBI. Furthermore, care was taken in the e-beam design to minimize low-intensity tails in the x-ray source spot and in the thermal design to minimize spot movements, since both are known to reduce spatial coherence and, thus, contrast^22^. For the experiments described below, primarily the emission in the 15-35 keV energy interval is of relevance. Typically, sub-15-keV photons contribute more to dose than to image contrast due to high absorption in the sample and we therefore use a 210 μm Al filter to reduce the low-energy emission of the source. The emitted Al-filtered spectrum is shown in Fig. 1b. The higher-energy photons (>35 keV) interact to a lesser degree with the sample and are in addition detected with low efficiency. In this 15-35 kV range the full flux is 3.5 × 10^12^ ph/(s × sr) and the corresponding relevant brightness is 7.0 × 10^10^ ph/(s × mm^2^ × mrad^2^). The source and system parameters are described in more detail in the Methods section and in the Supplementary Material.

### High-resolution short-exposure absorption tomography of a mouse

**Figure 2:**
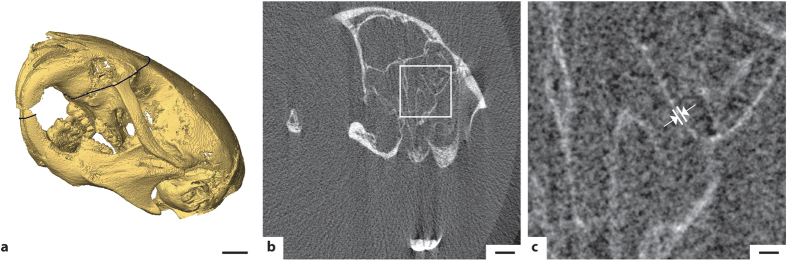
High-resolution absorption CT of mouse head. The total exposure time was 73 seconds and the total dose 400 mGy. **(a)** Surface rendering of the mouse skull based on the absorption tomography. Scale bar, 2 mm. **(b)** Slice through the mouse skull as indicated by the black line in (a). Scale bar, 1 mm. **(c)** Detail of the slice showing several 25-40 μm structures. Arrows indicate 34 μm FWHM example. Scale bar, 200 μm.

The arrangement allows high-spatial resolution absorption CT of rodents. Figure 2a shows the 3D rendering of the mouse head from a whole-animal scan reconstructed with 7.6-μm isotropic voxels. The image provides a high degree of detail as evidenced by structures in the teeth and the bone structure. Figure 2b shows a tomographic slice through the skull and Fig. 2c a zoom-in of part of the slice. Several 3-5-pixel (25–40 μm) bone structures are observable in the images. The observable detail and spatial resolution is estimated from the intensity patterns of sharp edges in the image, cf. knife edge scans^25^. Edge scans of the bone structures in Fig. 2b and c typically exhibit a 25–75% intensity rise of approx. 25 μm, from which we estimate a half-period resolution of approx. 25 μm, which is consistent with our observations above. The total exposure time was 73 seconds (121 projections ×0.6 s, in steps of 1.5° over 180°). The magnification was 1.19 and the dose 400 mGy. In the Supplementary Material surface renderings and tomographic slices based on reconstructions from 1-, 6- and 30-minute total exposures are depicted for comparison.

### High-resolution phase-contrast tomography of a phantom

**Figure 3:**
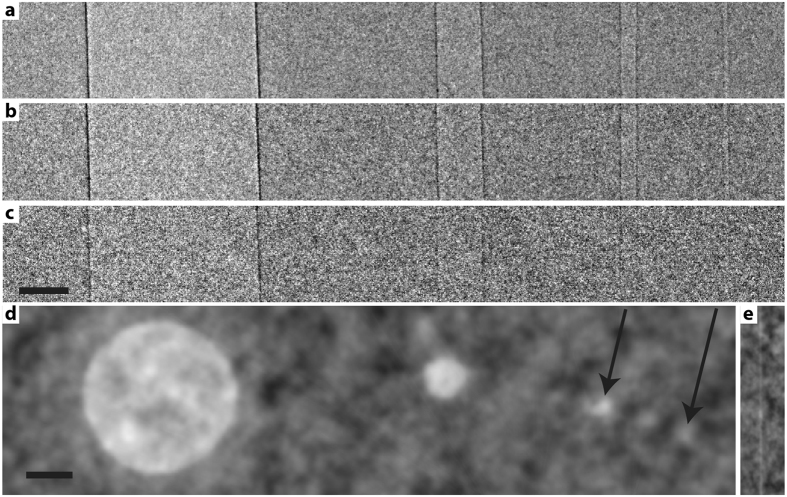
Phase contrast imaging of angiography/lung phantom. The 22 mm diameter water/plastic phantom contains four air-filled tubes of inner diameters 684, 176, 50 and 23 μm. The three top images show projection imaging at *M* = 4.2 with **(a)** 120 s exposure time/229 mGy dose, **(b)** 60 s/114 mGy and **(c)** 12 s/23 mGy. The lower image **(d)** shows a tomographic reconstruction. Arrows show smallest vessels. **(e)** Sagittal view of the 23 μm vessel in the tomographic dataset. Scale bar, 200 μm.

Our angiography/lung phantom mimics CO_2_-filled blood vessels or air-filled lung structures in a mouse-size object, where the gas-filled vessel structures range from 23 to 684 μm diameter. Figure 3 shows the result of propagation-based phase-contrast imaging. Figure 3a–c show projection imaging at *M* = 4.2 magnification with different exposure time and dose, from (a) 120 s/229 mGy over (b) 60 s/114 mGy to (c) 12 s/23 mGy. It is interesting to note that the 23 μm vessel is observable at the 60 s/114 mGy exposure while for 12 s/23 mGy the smallest observable vessel diameter is the 176 μm. This assessment of the observable detail is supported by calculations of the signal-to-noise ratio (SNR). Table S1 in the Supplementary Material shows the results. Figure 3d shows a tomographic reconstruction of the gas-filled structures. Here we used 6 minute total exposure time (180 projections ×2 s, in steps of 1° over 180°) and *M* = 3. All gas filled vessels are observable in this 686 mGy exposure, where the smallest vessel is clearly visible from examining several adjacent slices. Figure 3e shows a sagittal view of the smallest vessel. From these experiments we conclude that our system allows high-spatial-resolution phase-contrast imaging (few tens of μm) in soft tissue/gas structures with very short exposure times (approximately a minute) and at dose levels acceptable for *in-vivo* rodent studies.

### High-resolution phase-contrast tomography of mouse lungs

**Figure 4:**
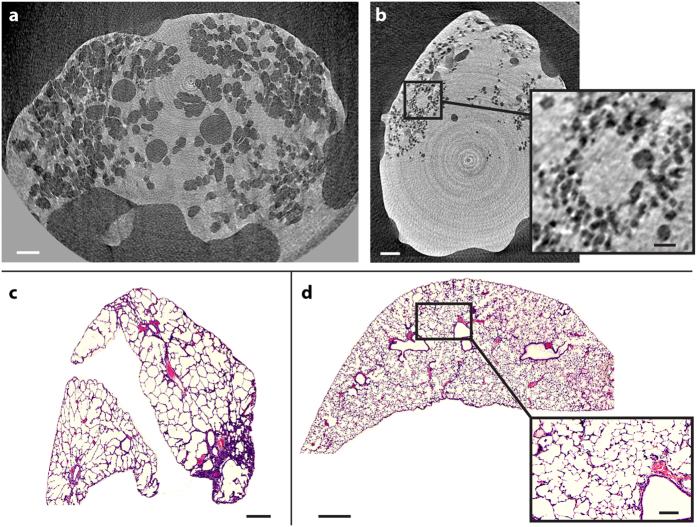
Mouse lung propagation-based phase-contrast tomography. **(a)** Slice of a lung with pulmonary emphysema from a 6-minute scan. Scale bar, 1 mm. **(b)** Slice of healthy lung, showing smaller structures than for the lung with emphysema. Scale bar, 1mm. The insert shows the alveolar structure in more detail. Scale bar, 250 μm. **(c)** Comparative histology of the lung with emphysema. Scale bar, 500 μm. **(d)** Comparative histology of the healthy lung. Scale bar, 500 μm. Insert shows more detail. Scale bar, 100 μm.

Figure 4 depicts the high-resolution tomographic imaging of two excised air-filled mouse lungs, one with pulmonary emphysema (a) and one healthy control (b). The air-filled bronchi and alveoli are black and the soft tissue of the lung is light gray. The lungs are surrounded by air (black) and the control lung is not completely filled with air. It is clear that the alveolar structures of the emphysematous lung are much larger than those of the healthy control, as expected. The inset in Fig. 4b shows a magnified view of the alveoli in the healthy lung. In this slice, air-filled structures (alveoli) with diameters <50 μm are observed. Edge scans of the alveoli boundaries typically exhibit 25–75% rise of ∼28 μm, suggesting to a half-period resolution of <30 μm. The total exposure time for each sample was 6 minutes (180 projections ×2 s, in steps of 1° over 180°). The magnification was 1.67 and the dose 2.6 Gy. In the Supplementary Material the same sample is reconstructed from a 30-minute exposure, (900 projections ×2 s, in steps of 0.2° over 180°; 13 Gy) data for comparison. Here we observe <40 μm diameter alveoli and the estimated half-period resolution is <20 μm (25–75% rise is 18 μm). We note from the introduction that the higher doses used here are appropriate for imaging excised samples. The Supplementary Material also includes a video surface rendering of an emphysematous lung, indicating the high contrast in the data and the extra structural information obtained from tomography.

### Comparison with histology

After the scan, the lung samples were embedded, sliced, and stained for histology. The results are shown in Fig. 4c and d. In general, the PBI tomography data can be concluded to be in good agreement with the histology, proving that we in fact detect individual alveoli with high contrast. The typical alveoli diameters in the emphysematous lung is in the 200-μm range (with a considerable spread), both in CT and in histology, while in the healthy lung the typical diameter is in the 50–60 μm range. The numbers are consistent with recent experiments at synchrotrons with healthy mouse lungs^26^.

## Discussion

We have demonstrated that small-animal imaging can be performed with high spatial resolution and at reasonable exposure time and acceptable dose with a laboratory system, both with absorption contrast (for bone imaging) and with phase contrast (for soft tissue imaging). The method relies on a magnifying propagation-based arrangement and a high-brightness electron-impact microfocus source. The arrangement allows for rapid and simple change of parameters to optimize resolution, contrast and signal-to-noise ratio for each imaging situation.

The absorption-contrast imaging of a whole mouse demonstrates that few-ten-μm details can be observed with minute-range exposure times and a 400-mGy dose. It is interesting to note that the magnifying arrangement necessary for the PBI also benefits the absorption imaging when a small-spot high-brightness source is used since the resolution limitation due to the detector point-spread function (here 27 μm full width at half maximum) can be overcome. For the phase-contrast imaging it is encouraging to observe few-tens of μm gas/tissue structures with minute exposure time and acceptable dose, 100 mGy in the phantom. For comparison, present typical live mouse imaging is performed with a few-100 mGy dose while up to a Gy may be used for special purposes^2^. As stated above, propagation-based imaging (PBI) was chosen before grating-based imaging (GBI) due to PBI´s dose efficiency and lower scan times despite that phase retrieval will be more complex for realistic multi-material objects. Fortunately algorithms handling such situations are presently emerging^27^.

The high-resolution short-exposure-time laboratory PBI imaging of gas/tissue interfaces has important applications, both for small-animal imaging and for 3D histology-like examinations of organs. As for laboratory phase-contrast lung imaging, elegant previous work based on the integrated (low-spatial resolution) dark-field scattering signal from a GBI system has successfully demonstrated discrimination between healthy and diseased lungs, with pulmonary emphysema^28^,^29^. The method shown in the present paper provides detailed imaging data of the 3D lung structure from the organ to close to the cellular scale. Such data is valuable for improved multiscale lung modelling^30^ and quantitative measures of, e.g., surface areas and alveolar density may be extracted for a better understanding of the state of the healthy and diseased lung without the need for classical histology^31^. It also allows for a detailed assessment of the structural changes associated with, e.g., pathological states or lung development, as presently demonstrated by synchrotron-based experiments with high dose^26^,^32^,^33^. In addition, CO_2_ angiography of tumor microvasculature may become important in angiogenesis research^34^.

As for the source, the brightness of the 400 W electron-impact liquid-metal jet source in the energy range relevant for small-animal imaging (15–35 keV) exceeds the brightness of the compact accelerator-based sources several times, resulting in imaging with significantly shorter exposure-times. In addition, the x-ray emission angle of the electron impact tubes is large compared to the typically few-mrad emission from accelerator-based sources, thereby allowing for compact magnifying arrangements for high-resolution whole-animal imaging, both in absorption and for PBI. Although the monochromatic emission of accelerator-based sources^14^ may be favorable in certain applications, simulations of the present experiments using our in-house software^35^ show a negligible difference in image quality for comparable dose when a monochromatic source is used instead of the actual source spectrum. Finally, we note that that the liquid-jet electron-impact tubes are significantly less complex than their accelerator-based alternatives and, thus, easier to integrate in small-animal imaging equipment.

In summary, we conclude that the methodological advances demonstrated here for absorption-based as well as propagation-based phase-contrast imaging opens up for imaging bone and soft-tissue structures with cellular spatial detail in whole-body small-animal objects at acceptable dose and exposure time. Furthermore, the present 400 W e-beam power and 8 μm spot size operation of the source is far from its theoretical limits, making future increases in source power and brightness highly realistic. Possibly, exposure times can be reduced >10 times, making, e.g., gated kinematics studies presently requiring a synchrotron sources^36^ feasible also in the laboratory.

## Methods

### Laboratory x-ray tomography arrangement for high resolution and short exposure time

Figure 1 depicts the experimental arrangement with its x-ray source, sample and detector. The microfocus source is an electron-impact liquid-metal-jet source based on a prototype platform from Excillum AB, Sweden, using a Galinstan alloy (Ga-In-Sn, 68.5%:21.5%:10%) as anode jet material. The emission is filtered by 210 μm Al to reduce the low-energy radiation, including the Ga K_α_ and K_β_ line emission at 9.3 and 10.3 keV, that contributes more to dose than to image contrast via significant sample absorption. The 15–35 keV x-ray spectrum relevant for the imaging is dominated by the broad bremsstrahlung and the K_α_ and K_β_ line emission from In and Sn at 24.2 and 27.3 keV, and 25.2 and 28.5 keV, respectively. The sample is placed on a rotation stage. The 36 × 24 mm^2^ and 4008 × 2671 pixel CCD detector (FDI-VHR, Photonic Science, United Kingdom) has a 15 μm thick Gadox (Gd_2_O_2_S:Tb) scintillator, a pixel pitch of 9 μm, and a measured point spread function with a full width at half maximum (FWHM) of 27 μm.

The short-exposure, high-resolution imaging in high-magnified systems demonstrated here requires a high-power source with a spatially stable and small x-ray spot. Compared to commercially available liquid-jet micro-focus sources^37^, the prototype liquid-jet source used in the present paper is operated at a significantly increased electron-beam power and while still keeping the spot size small and spatially stable. The LaB_6_ cathode generates a 400 W, 50 kV electron beam which is focused onto the 250 μm diameter metal jet by a magnetic lens in combination with alignment and deflection coils, generating a high-quality x-ray spot with a FWHM of 8 μm and very limited low-intensity tails. For comparison, in our previous small-spot (<8 μm) high-resolution imaging we typically operated at 30–40 W electron beam power (cf. e.g., Refs 21, 22.) The stable operation of the present small-spot/high-power source was enabled by increased water cooling for improved thermal stability and a bent e-beam column to protect the cathode from anode vapor by removing the line-of-sight between anode and cathode. A well-defined and stable x-ray spot is important for high image contrast, especially in high-spatial-resolution PBI but also in absorption imaging. The spot size was measured with a 100 nm outermost zone-width Au zone plate and the quantitative spectrum (cf. Fig. 1b) was measured with a CdTe spectrometer (X-123, Amptek Inc., US). Operating at 400 W, the emitted flux in the imaging-relevant 15–35 keV bandwidth (BW) is 3.5 × 10^12^ photons/(s × sr) and the corresponding 15–35-keV-BW brightness is 7.0 × 10^10^ photons/(s × mm^2^ × mrad^2^). For comparison, the 3%-BW brightness around the 24.2 keV In line K_α_ is 1.8 × 10^10^ photons/(s × mm^2^ × mrad^2^ × 3%BW).

### Whole-body mouse

The 12-week mouse (strain BrafMycER KI/KI) was euthanized and chemically fixed in 4% paraformaldehyde (PFA) in phosphate buffered solution and subsequently embedded in 3% agarose gel (Sigma-Aldrich, CAS 39346-81-1) inside a polypropylene tube. The animal experiment was carried out in accordance with the Animal Protection Law (SFS 1988:534), the Animal Protection Regulation (SFS 1988:539), and the Regulation for the Swedish National Board for Laboratory Animals (SFS 1988:541) and approved by the Stockholm regional ethics committee for animal research (N283/12).

### Angiography/lung phantom

The phantom consists of 4 air-filled vertical low-density polyethylene (LDPE) tubes with inner diameters 23 μm, 50 μm, 176 μm, and 684 μm placed in a water-filled cylindrical PMMA holder of 16 mm inner diameter and 22 mm outer diameter. This corresponds in absorption to about 21 mm of tissue, a typical object thickness in mouse imaging. LDPE was chosen because of its density, which is similar to that of water and tissue, thus making the tubes a proper representation of a CO_2_-filled blood vessels^21^ or air-filled lung structures^28^. Although the phantom correctly represent the image SNR of gas-tissue structures it naturally does not include the more complex background from e.g., bone, hair, and movements in a live mouse.

### Mouse lungs and the pulmonary emphysema protocol

The excised lungs came from 6- to 8-week old pathogen-free female C57BL/6 N (Charles River Laboratories, Sulzfeld, Germany) mice. For the induction of pulmonary emphysema, a solution of pancreatic elastase in sterile phosphate-buffered saline was applied orotracheally (80 U per kilogram of body weight). Control mice received 80 μL sterile phosphate-buffered saline. Mouse lungs were excised 28 days after elastase application, inflated with air, tied at the trachea, and placed in a formalin-filled plastic container. There was approx. 2 weeks between the excise and the x-ray imaging, leading to some leakage of air in some cases. The lung experiments were performed with permission of the Institutional Animal Care and Use Committee of the Helmholtz Zentrum Munich and carried out in accordance with national (Gesellschaft für Versuchstierkunde—Society for Laboratory Animal Science) and international (Federation for Laboratory Animal Science Associations) animal welfare guidelines. The Institutional Animal Care and Use Committee of the Helmholtz Zentrum Munich approved all the experimental protocols.

### Data acquisition

The mouse absorption tomography of Fig. 2 was performed with a source-object-distance (*R*_1_) of 29.8 cm and a object-detector-distance (*R*_2_) of 5.7 cm, resulting a magnification of *M* = 1.19. The reconstruction is based on 120 projections each exposed 0.6 s and an angular step size of 1.5° over 180°, resulting in 73 s total exposure time and 400 mGy dose. In the Supplementary material we also show reconstructions with 180 × 2 s with 1° steps (6 minutes, 1.9 Gy), and 900 × 2 s with 0.2° steps (30 minutes, 9.5 Gy) for comparison.

The PBI phase-contrast projection imaging of the phantom in Fig. 3 was performed with *M* = 4.2 (*R*_1_ = 60 cm and *R*_2_ = 194 cm). Exposure time and dose was 120 s/229 mGy, 60 s/114 mGy and 12 s/23 mGy (Fig. 3a–c). The PBI tomography of the phantom in Fig. 3d had *M* = 3 (*R*_1_ = 60 cm and *R*_*2*_ = 120 cm) and 180 projections ×2 s, angular steps of 1° and 686 mGy dose.

The PBI phase-contrast lung tomography in Fig. 4 was performed with *M* = 1.67 (*R*_1_ = 30 cm and *R*_2_ = 20 cm). The reconstructions are from 180 projections×2 s with an angular step of 1° (6 minutes, 2.4 Gy). In the Supplementary material we show reconstructions from 900 projections×2 s with 0.2° step (30 minutes, 9.5 Gy).

### Data processing and reconstruction

All experimental data was processed with the same procedure. The projections were first flat-field corrected and then phase-retrieved using the Paganin method^38^ before the tomography. The phase-retrieval assumed the appropriate constants for each experiment, i.e., bone/soft tissue (Fig. 2), water/air (Fig. 3) and tissue/air (Fig. 4). We note that the phase-retrieval step had a negligible influence on the absorption imaging of Fig. 2. The tomographic reconstruction was performed with the cone-beam-corrected filtered back projection in the Octopus software (Inside Matters, Aalst, Belgium) with 7.6 μm voxel size. The 3D surface rendering employed the Amira software (Visage Imaging, San Diego, CA, US) on 2 × 2 binned data.

### Histology

The lungs were washed to remove paraformaldehyde and then dehydrated and embedded in paraffin. At an interval of 0.5 mm, multiple 10-μm-thin slices were prepared in the coronal plane. The slices were stained by using the Mayer hematoxylin-eosin staining routine protocol. Subsequently, the slices were scanned at 2.5× and 20.0× magnifications to create digital images.

## Additional Information

**How to cite this article**: Larsson, D. H. *et al*. High-resolution short-exposure small-animal laboratory x-ray phase-contrast tomography. *Sci. Rep.* **6**, 39074; doi: 10.1038/srep39074 (2016).

**Publisher's note:** Springer Nature remains neutral with regard to jurisdictional claims in published maps and institutional affiliations.

## Supplementary Material

Supplementary Material

Supplementary Video 1
