# Supplementary material for: High-resolution short-exposure small-animal laboratory x-ray phase-contrast tomography
Source: Sci Rep. 2016 Dec 13;6:39074. doi: 10.1038/srep39074 (PMC5153650; doi:10.1038/srep39074)
Supplement: Supplementary Material [file srep39074-s1.pdf]

Supplementary material

High-resolution short-exposure small-animal laboratory x-ray phase-contrast tomography

Daniel H. Larsson,[1] William Vågberg,[1] Andre Yaroshenko,[2] Ali Önder Yldirim[3], and Hans M. Hertz[1]

[1] Department of Applied Physics, KTH Royal Institute of Technology/Albanova, 106 91 Stockholm, Sweden

[2] Physik-Department & Institut für Medizintechnik, Technische Universität München, Garching, Germany.

[3] Institute of Lung Biology and Disease, Member of the German Center for Lung Research (DZL), Helmholtz Zentrum München, Neuherberg, Germany.

*Correspondence: hertz@biox.kth.se

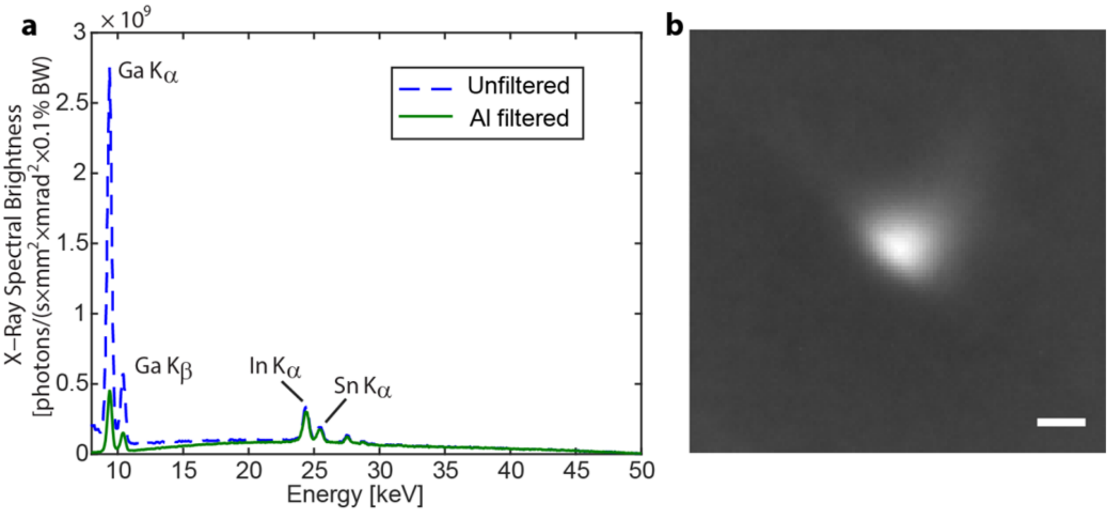

Figure S1. X-ray source properties. (a) Liquid-metal-jet source brightness with and without the 210 μm Al filtering when operated at 400 W and 8 μm spot size. It is clear that a thicker Al filter would reduce the dose-contributing 9 keV peaks even further but at the expense of longer exposure time since it also would reduce the 15-35 keV flux essential for the imaging. (b) Zone-plate image of the source spot showing the 8 μm FWHM spot with short tails. Scale bar, 5 μm.

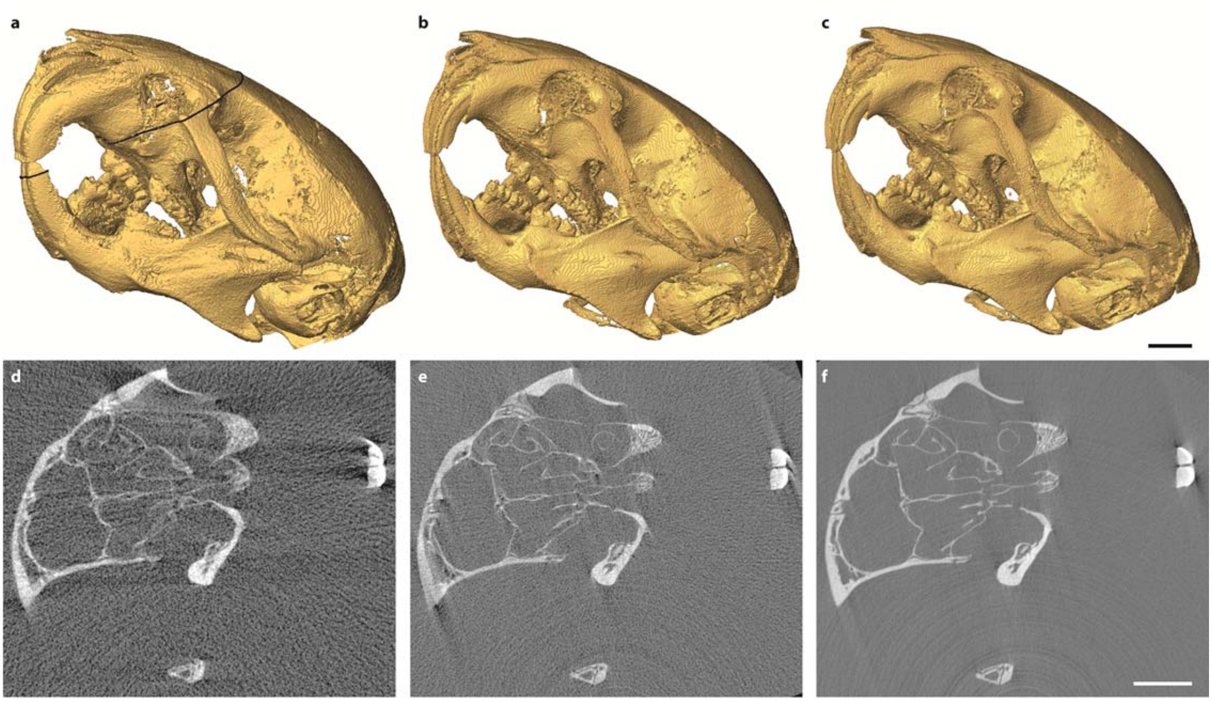

Figure S2. Comparison of image quality in mouse skull absorption tomography. Here we show surface rendering of the mouse skull and a selected tomographic slice from three experiments with different exposure times. (a) and (d) 73 second exposure time (120 projections x 0.6 s, 1.5°/projection, over 180°) and 400 mGy dose . (b) and (e) 6-minute exposure time (180 projections x 2 s, 1.0°/projection, over 180°) and 1.9 Gy. (c) and (f) 30-minute exposure time (900 projections x 2 s, 0.2°/projection, over 180°) and 9.4 Gy. Black scale bar for (a-c), 2 mm. White scale bar for (d-f), 2 mm. The black line in (a) indicates the position of the tomographic slices in (d-f).

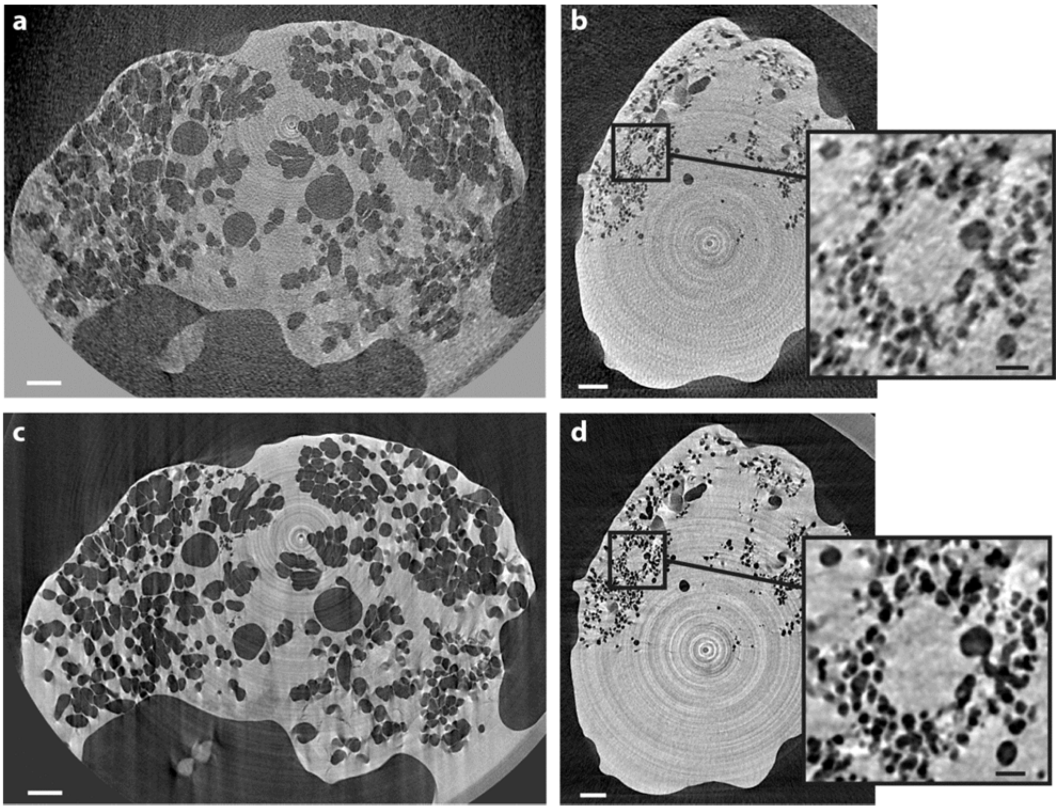

Figure S3. Comparison of image quality in PBI lung imaging. Upper row: 6 minute exposure time (180 projections x 2 s, 1.0°/projection, over 180°; 2.6 Gy) of sick (a) and healthy (b) lungs. Lower row: 30 minute exposure time (180 projections x 2 s, 0.2°/projection, over 180°; 13 Gy) of the same sick (c) and healthy (d) lungs. Here the longer exposures produce a clearer image. White scale bars, 1 mm. Black scale bars, 250 μm.

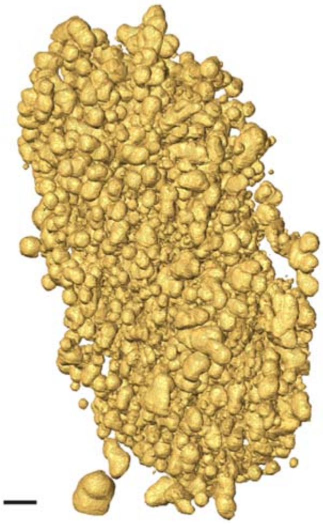

Figure S4. Alveolar structure in excised air-filled lung with emphysema. Surface rendering of lung alveolar structure from a 20 minute, 7.2 Gy exposure. Scale bar, 500 μm. See also video.

	Diam=684 µm	Diam=176 µm	Diam=50 µm	Diam=23 µm
**Fig 2a: 120 s**	127	52	26	17
**Fig 2b: 60 s**	92	36	18	10
**Fig 2c: 12 s**	39	15	6.7	3.8

Table S1. Signal-to-noise ratio (SNR) in the Fig. 3a-3c phantom projection imaging.
We use the differential signal-to-noise ratio as a quantitative measure on what objects can be detected in the projection images of our angiography/lung phantom. The SNR of each tube is calculated for a length of the tube equal to its width according to equation (7.6) in Ref. 35, which is analogous to equation (3.2) in Ref. S1,

$$\mathrm{SNR}^2 = m\,\frac{(S - S_{bg})^2}{\sigma_{bg}^2},$$

where m is the extent of the object in number of pixels, S is the average pixel value of the object, S_{bg} the average background intensity, and σ_{bg}^2 the standard deviation of the background. The detectability limit is typically set to SNR≈5 (cf. the Rose criterion). We note that this limit is approximate by definition, and that the actual experimental limit is also influenced by, e.g., observer and noise properties.

Reference

S1. Jacob Beutel, Harold L Kundel, and Richard L Van Metter. *Handbook of medical imaging, volume 1: physics and psychophysics*. SPIE Press Bellingham, WA, 2000.

Video 1. Alveolar structure in excised air-filled lung with emphysema. Surface rendering of lung alveolar structure from a 20 minute, 7.2 Gy scan.
